# Environmental factors of diarrhea prevalence among under five children in rural area of North Gondar zone, Ethiopia

**DOI:** 10.1186/s13052-018-0540-7

**Published:** 2018-08-16

**Authors:** Atalay Getachew, Alebachew Tadie, Mulat G.Hiwot, Tadesse Guadu, Daniel Haile, Teklay G.Cherkos, Zemichael Gizaw, Marta Alemayehu

**Affiliations:** 1 College of Medicine and Health Sciences, Institute of Public Health, Department of Environmental & Occupational Health & Safety, University of Gondar, P.O. Box 196, Gondar, Ethiopia; 2 Department of Ecological and Systematic Zoology, College of Natural and Computational Science, University of Gondar, Gondar, Ethiopia; 3 Department of Medical Microbiology, Institute of Biomedical Sciences, University of Gondar, Gondar, Ethiopia

**Keywords:** Diarrhea, Environmental factors, Under-five children, North Gondar zone

## Abstract

**Background:**

In the sub-Saharan countries, many of the children including found in health facilities are not having sufficient care of diarrhea. Diarrheal disease in these countries is one of the main causes of deaths for under-five children.

**Methods:**

A community-based cross-sectional study design was used to collect data from May–June, 2016 to determine environmental factors of diarrhea prevalence among under five children in North Gondar Zone. Structured interview questionnaire and observational checklist were used to collect data. Using probability proportion to size, number of households was determined in each district. The multivariable binary logistic regression analysis with a 95% confidence interval and *p* < 0.05.was used to identify environmental factors associated with childhood diarrheal disease.

**Results:**

Of the total 736 individuals surveyed from 736 households, a total of 163 (22.1%) with [95% CI (19.1–25.1)] under –five children had 2 week period diarrhea prevalence. Roof material [AOR: 1.99, 95% CI (1.1–3.82)], hand washing facility [AOR: 0.52, 95%CI (0.33–0.82)], presence of Latrine facility [AOR: 1.65, 95% CI (1.01–2.72)], presence of feces around the pit hole [AOR: 1.65, 95% CI (1.01–2.72)], presence of feces around the house compound [AOR: 1.65, 95% CI (1.01–2.72)] and risk of contamination of household storage had significant associations with diarrheal morbidity.

**Conclusion:**

The prevalence of childhood diarrheal disease among under – five children in rural settings of northwest Ethiopia was high. Type of roof material, hand washing facility, presence of Latrine facility, presence of feces around the pit hole, presence of feces around the house compound and risk of contamination of household storage had significant associations with diarrheal morbidity.

## Background

In the sub-Saharan countries, many of the children including found in health facilities are not having sufficient care of diarrhea. Diarrheal disease in these countries is one of the main causes of deaths for under-five children [1]. One child passed away from the world within each 15 s due to diarrheal disease [2]. Moreover, in the developing countries alone about two millions of people most of which are under five children die annually [3]. Therefore, there should be an increased work at health care facilities and among the community for the childhood diarrhea care quality [1].

Many factors are responsible for the diarrheal disease. They are socio-economic [4, 5], behavioural like breastfeeding [6, 7] and environmental factors such as water, sanitation and waste disposal mechanism [8–11]. Different studies have been conducted in different parts of Ethiopia on diarrhea prevalence and reported 12.2, 35.6, 22.5, 9.9, and 28.9% [6–10], respectively.

Environmental factors like the type of water source, presence of sanitation facilities, solid waste disposal system and floor type in the kitchen are found to be crucial contributors for the high prevalence of diarrheal diseases. Particularly, diarrhea occurrence is more associated with unsafe/unprotected water sources e.g. ponds, wells, rivers, lakes [12]. Other more environmental health risk factors of childhood diarrhea include improved sanitation, hand washing facilities, poor knowledge on diarrheal cases, and improved latrine [8, 11]. Therefore, the current study was designed to determine the prevalence of diarrheal disease in under five children and assess the associated environmental factors in the rural areas of North Gondar zone, Ethiopia.

## Method

### Study area

A community-based cross-sectional study was conducted in North Gondar Zone from April to June 2016. Gondar town is located 739 km far from Addis Ababa to the Northwest of Ethiopia and 180 km in the north direction of Bahir Dar (Capital of Amhara Region). North Gondar is one of the eleven zones in Amhara Regional State having 22 administrative districts. As the data gained from North Gondar Zonal Health Department, the total projected population of North Gondar Zone in 2015/16 is 3,704,740. The majority of which 2,920,007 (78.8%) populations reside in rural areas whereas the rest 784,733 (21.2%) are in urban areas.

### Study design and period

A community based cross-sectional study design was used to collect data from May–June 2016.

### Sample size determination and sampling procedures

Epi info version 3.5.3 was used to calculate the sample size based on an assumption that 18% of the under-five children had two-week prevalence of diarrhea in North Gondar (26) with marginal error of 4%, a standard score corresponding to 95% certainty, design effect of 2, accounted for two-stage sampling and none response rate 5%. The total sample size that was included in the study is 743 households that should have at least one under-five child. From randomly selected four districts (Dembia, Gondar Zuriya, Chilga, and Sanja) of the 22 total districts of North Gondar Zone, multi-staged sampling procedure was employed.

Using probability proportional (PPS) to size, the number of households was determined in each district. Then, 25% of total kebeles was selected from each district by simple random sampling technique and systematic sampling technique was applied to select study households. In case, where there are more than one under-five children in the same household, index child was selected by lottery method to collect information on child’s health characteristics. The first household interview was identified by a modified random walk method and if there is no mother/care taker or under five child in the selected household, the next nearest household was included in the survey.

### Data collection methods and tools

The data were collected by using face to face interview with pretested structured questionnaires and observational checklists which were prepared in English and translated to the local language, Amharic. To improve the quality of data collection, two-day training was given to 16 data collectors and 8 supervisors about way of interview, the nature of questionnaires, rechecking of filled questionnaires and approaches to household heads.

### Data analysis

All data collection forms were checked for completeness and reliability before entry into software. Data entry and cleaning were done using Epi info version 3.5.3 computer software. Data were analyzed by using SPSS ver.20. Descriptive analyses were deployed for both dependents and independent variables. The frequency distribution of both dependent and independent variables were worked out. Logistic regression analysis was used to see the relationship of selected variables to diarrheal disease. Eight variables with *P*- value less than 0.2 in bivariate analysis were included in the multivariate logistic regression. Finally, data were presented in the form of tables and figures.

### Ethical considerations

Ethical clearance was obtained from the Institutional Ethical Committee of the University of Gondar. Moreover, the consent of participants was obtained from the respective district health offices. The confidentiality of information was maintained during and after an interview in which coding was used for all the data collected. Participants in the study had given verbal consent.

## Results

### Socio-demographic characteristics of the study participants

In this study, a total of 736 respondents of households participated whose mean (±SD) age was 30 ± 7 years nearly half, 362 (49.2%) respondents were aged between 25 and 34 year. The majority, 690 (93.8%) of participants religion were orthodox and more than half of the respondents 431 (58.5%) were unable to read. Most of the respondents 693 (94.2%) were married and housewives 632 (85.8%). Majority, 463(62.9%) of the respondents had a family size less than five individuals and 423 (57.3%) of the respondent had income less than 1000 ETB (Table 1).

**Table 1:** Socio-demographic characteristics of the study participants in North Gondar Zone, June, 2016 (*N* = 736)

Variables	Number	Percent (%)
Age		
< 15	3	0.4
15–24	149	20.2
25–34	362	49.2
> 35	222	30.2
Religion of parents		
Orthodox	690	93.8
Protestant	6	0.8
Muslim	40	5.4
Educational level		
Unable to read and write	431	58.6
Read and write	62	8.4
1–8	135	18.3
9–12	75	10.2
> 12	33	4.5
Marital Status		
Married	693	94.2
Single	2	0.3
Divorced	35	4.8
Widowed	8	0.8
Occupation of the mother		
Government employee	24	3.3
Housewife	632	85.8
Merchant	35	4.8
Farmer	45	6.1
Family size		
≤ 5	463	62.9
> 5	273	37.1
Income		
< 1000 Birr	423	5.3
1000–1999 Birr	284	25.8
2000–2999Birr	21	47
> 3000 Birr	8	7

### Environmental characteristics

Out of the 736 respondents interviewed, majority of respondents 562(76.4%) had less than two rooms per household whose most of living room floor and roof type is mud 725(98.5%) and corrugated iron 667(90.6%) respectively. Only 173(23.5%) of the household have separate house for animals. Most of the households, 395 (82.0%) used the Pit latrine without slab and only half of the households, 396 (53.8%) used piped water source for drinking. From sanitary risk assessment survey, majority of the source water, 356 (48.4) and household storage, 314 (42.7) had medium risk of contamination. Moreover, the majority of households 556 (75.5%) used open field solid waste disposal (Table 2).

**Table 2:** Environmental characteristics of respondent household in North Gondar Zone, June, 2016 (*N* = 736)

Variables	Number	Percent (%)
Type of roof material		
Thatched	69	9.4
Corrugated iron	667	90.6
Type of floor material		
Mud	725	98.5
Cement	11	1.5
Number of rooms per household		
< =2 rooms	562	76.4
> 2 rooms	174	23.6
Animals live with human		
Yes	173	23.5
No	563	76.5
Presence of Latrine facility		
Yes	482	65.5
No	254	34.5
Type of Latrine facility (*N* = 482)		
Flush/pour flush latrine	14	2.9
Ventilated improved pit latrine	39	8.1
Pit latrine with slab	34	7.1
Pit latrine without slab	395	82
Ownership of latrine (*N* = 482)		
Private	369	76.6
Shared	113	23.4
Presence of feces around the pit hole (*N* = 482)		
Yes	56	7.6
No	426	57.9
Presence of feces around the house compound		
Yes	112	15.2
No	624	84.8
Source of drinking water		
Piped water	396	53.8
Orotected spring and well	190	25.8
Unprotected spring and well	133	18.1
River	17	2.3
Intermittent of Water Supply		
Yes	461	62.6
No	275	37.4
Consumption in liter/capita/day		
< 20 L/Capita/day	675	91.7
> =20 L/Capita/day	61	8.3
Household water treatment		
Boiling	13	1.8
Filtering	19	2.6
Use of chemicals	40	5.4
Allowing water to settle	12	1.6
No treatment	652	88.6
Risk of contamination of Household storage		
Low	206	28
Medium	314	42.7
High	198	26.9
Very high	18	2.4
Risk of contamination of source water		
Low	306	41.6
Medium	356	48.4
High	74	10.1
Hand washing facility		
Yes	392	53.3
No	344	46.7
Place of solid waste disposal		
Pit	168	22.8
Open field	556	75.5
Burning	12	1.6

### Prevalence of diarrhea

From a total of 736 under five children, 163 under – five children had diarrhea in the 2 week period prior to data collection. Therefore, the 2 week period prevalence of diarrhea was found to be 22.1% [95% CI (19.1–25.1)].

### Environmental factors associated with diarrheal disease

In the Univariable binary logistic regression analysis type of roof material, hand washing facility, presence of Latrine facility, ownership of latrine, intermittent water supply, household water treatment, presence of feces around the pit hole, presence of feces around the house compound, risk of contamination of household storage had a p - value less than 0.2 and further analyzed by multivariable binary logistic regression. Finally type of roof material, hand washing facility, presence of Latrine facility, presence of feces around the pit hole, presence of feces around the house compound and risk of contamination of household storage had significant associations with diarrheal morbidity.

Children whose household roof material thatched had two times higher odds of diarrhea than children whose household roof material is corrugated iron [AOR: 1.99, 95% CI (1.1–3.82)]. The risk of developing diarrhea in children who had hand washing facility was 48% lower chance [AOR: 0.52, 95%CI (0.33–0.82)] compared to their counterparts (Table 3).

**Table 3:** Environmental factors associated with diarrheal disease prevalence among under five children in North Gondar Zone, June, 2016

Variable	Diarrhea prevalence		COR (95% CI)	AOR (95% CI)
	Yes, n (%)	No, n (%)		
Type of roof material				
Thatched	20(29.0)	49(71.0)	1.49(0.86–2.59)	1.99(1.1–3.82)*
Corrugated iron	143(21.4)	524(78.6)	1.00	1:00
Number of rooms in the house				
< =2 room	124(22.1)	438(77.9)	0.98(0.65–1.47)	
> 2 room	39(22.4)	135(77.6)	1:00	
Hand washing facility				
Yes	63(18.3)	281(81.7)	0.66(0.46–0.93)*	0.52 (0.33–0.82)*
No	100(25.5)	292(74.5)	1.00	1.00
Animals live with human				
Yes	38(22.0)	135(78.0)	0.99(0.65–1.49)	
No	125(22.2)	438(77.68	1.00	
Presence of Latrine facility				
Yes	100(20.7)	382(79.3)	1.00	1:00
No	63(24.8)	191(75.2)	1.26(0.88–1.81)	1.65(1.01–2.72)*
Type of latrine facility				
Unimproved	84(21.3)	311(78.7)	1.20(0.66–2.17)	
Improved	16(18.4)	71(81.6)	1:00	
Ownership of latrine				
Private	83(22.5)	286(77.5)	1.00	1:00
Shared	17(15.0)	96(85.0)	0.61(0.34–1.08)	1.09(0.58–2.07)
Source of drinking water				
Unimproved	33(22.0)	117(78.0)	0.98(0.64–1.53)	
Improved	130(22.2)	456(77.8)	1.00	
Intermittent water supply				
Yes	114(24.7)	347(75.3)	1.52(1.04–2.20)*	1.23(0.79–1.91)
No	49(17.8)	226(82.2)	1.00	1.00
Water consumption in liter/capita/day				
< 20 L/Capita/day	153(22.7)	522(77.3)	0.669(0.332–1.349)	
> =20 L/Capita/day	10(16.4)	51(83.6)	1.00	
Household water treatment				
No	131(20.1)	521(79.9)	2.45(1.51–3.95)	0.67(0.37–1.21)
Yes	32(38.1)	52(61.9)	1.00	1.00
Presence of feces around the pit hole				
Yes	22(39.3)	34(60.7)	1.96(1.07–3.6)*	2.46(1.20–5.04)*
No	78(18.3)	348(81.7)	1.00	1.00
Presence of feces around the house compound				
Yes	48(42.9)	64(57.1)	3.32(2.17–5.08)**	2.09(1.24–3.54)*
No	115(18.4)	509(81.6)	1.00	1.00
Methods of solid waste disposal				
Pit	56(33.3)	112(66.7)	80,766(0.00-)	
Open field	107(19.2)	449(80.8)	38,494(0.00-)	
Burning	0(0)	12(100)	1:00	
Risk of contamination of Household storage				
Low	29(14.1)	177(85.9)	1:00	1:00
Medium	38(12.1)	276(87.9)	0.84(0.50–1.41)	1.07(0.61–1.89)
High	79(39.9)	119(60.1)	4.05(2.49–6.58)**	5.21(3.01–show [?A3B2 show $9#?]9.03)**
Very high	17(94.4)	1(5.6)	103.7(13.3–809.7)**	126.6(15.5–1036)**
Risk of contamination of source water				
Low	77(25.2)	229(74.4)	1:00	
Medium	71(19.9)	285(80.1)	0.74(0.51–1.07)	
High	15(20.3)	59(79.7)	0.77(0.41–1.41)	

* Statistically significant with *p*-value < 0.05, **statistically significant at *p*-value < 0.001

Children who had no latrine facility had two times higher odds of diarrhea than children who had latrine facility [AOR: 1.65, 95% CI (1.01–2.72)]. Presence of feces around the pit hole [AOR: 1.65, 95% CI (1.01–2.72)] and the house compound [AOR: 1.65, 95% CI (1.01–2.72)] had a significant association with diarrheal disease. The risk of developing diarrheal disease in children who had high sanitary risk of contamination of household storage had five times higher odds of diarrheal disease [AOR: 5.21, 95% CI (3.01–9.03)] compared to sanitary low risk of contamination of household storage. Moreover, children who had very high sanitary risk of contamination of household storage had strong statistically significant association [AOR: 126.6, 95% CI (15.5–1036)] with diarrheal disease (Table 3).

## Discussion

The current study determined the prevalence of diarrhea and assessed the environmental factors of diarrhea prevalence among under-five children. The prevalence of diarrhea in the current study was 22.1%. This result is higher than 9.9% in Sebeta town, Oromiya Region of Ethiopia [8] and 19.6% in a rural area of Shebedino district, southern Ethiopia [13] and it is close with the result 22.5% of east Ethiopia [9]. However, it is lower than 26.1% in Hadaleala District, Afar Region of northeast Ethiopia [5], 27.3% in Jigjiga district, Somali region of Ethiopia [12], 35.6% in Enderta Woreda, Tigray Region of Ethiopia [6] and 32.6% in Burundi [14].

In this study, type of roof material, hand washing facility, presence of Latrine facility, presence of feces around the pit hole, presence of feces around the house compound and risk of contamination of household storage had significant associations with diarrheal morbidity.

Children whose household roof material thatched had two times higher odds of diarrhea than children whose household roof material is corrugated iron. This might be due to the poor sanitation of the house in thatched roof compared to the corrugated iron.

The risk of developing diarrhea in children who had hand washing facility was 48% lower chance compared to their counterparts. This finding is similar with studies done in Sebeta town of Ethiopia [15] and also in Adama District Rural Kebeles [16]. The hand washing facility is important to mothers to easily wash their hands at critical times during the day which is important to reduce fecal-oral transmission of disease.

Children who had no latrine facility had two times higher odds of diarrhea than children who had latrine facility. This finding is in agreement with studies done in West Gojam Ethiopia [17] and in Deresha district, Southern Ethiopia [18]. The presence of latrine increases the chance of its utilization which in turn facilitates the safe disposal of feces. This is one way of decreasing contact between causative organisms of diarrhoeal and the host.

Presence of feces around the pit hole had a significant association with diarrheal disease. This result is comparable with studies done in Nekemte town, western Ethiopia [10] and in Addis Ababa, Ethiopia [19]. Presence of feces around the house compound had a significant association with diarrheal disease. This is due to the fact pathogens in feces disposed in compounds near the house can contaminate the environment and the food eaten by children which leads to diarrheal disease [20–23].

The risk of developing diarrheal disease in children who had high sanitary risk of contamination of household storage had five times higher odds of diarrheal disease compared to sanitary low risk of contamination of household storage. Moreover, children who had very high sanitary risk of contamination of household storage had strong statistically significant association with diarrheal disease. This finding is similar with studies done in Nigeria [24].

The rest variables like type of latrine facility, ownership of latrine, source of drinking water, intermittent water supply, and household water treatment were not significant variables in this study. But, they were significant in the previous studies conducted in different regions of Ethiopia [8, 11]. These discrepancy might be due to the strengthen of Ethiopia’s Health Extension Program (HEP) from time to time that has created greater awareness of how to prevent communicable diseases such as malaria, tuberculosis, HIV/AIDS and waterborne disease like diarrhea to the community [25]. Moreover, the discrepancy can be due to difference in seasonal variation.

## Conclusion

In the current study, prevalence of diarrhea found to be high (22.1%). Type of roof material, hand washing facility, presence of latrine facility, presence of feces around the pit hole, presence of feces around the house compound and risk of contamination of household storage had significant associations with diarrheal morbidity.

## Abbreviations

AOR: Adjusted odd ratio
Cl: Confidence interval
COR: Crude odd ratio
ETB: Ethiopian birr
PPS: Probability proportion to size
WHO: World health organization
